# Reflection revisited: how physicians conceptualize and experience reflection in professional practice – a qualitative study

**DOI:** 10.1186/s12909-018-1218-y

**Published:** 2018-05-11

**Authors:** Elisa Bindels, Christel Verberg, Albert Scherpbier, Sylvia Heeneman, Kiki Lombarts

**Affiliations:** 1 Department of Educational Development and Research, Faculty of Health, Medicine and Life Sciences, Maastricht University, Maastricht, the Netherlands; 2 Professional Performance research group, Institute for Education and Training, Academic Medical Center (AMC-UvA), Amsterdam, the Netherlands; 3 ICLON, Leiden University, Graduate School of Teaching, Leiden, the Netherlands; 4 Department of Pathology, Faculty of Health, Medicine and Life Sciences, Maastricht University, Maastricht, the Netherlands

**Keywords:** Reflection, Reflective practice, Continuing medical education, Professional development, Qualitative research methods

## Abstract

**Background:**

For the purpose of continuous performance improvement, physicians are expected to reflect on their practice. While many reflection studies are theoretically oriented and often prescriptive in the sense that they conceptualize what reflection should look like, the current study starts with practicing physicians themselves and maps how these physicians conceptualize and experience reflection in daily professional practice.

**Methods:**

We conducted a qualitative study using in-depth interviews with 13 hospital-based physicians from various specialties and institutions. The interviews were transcribed verbatim and were analyzed iteratively, following the interpretative phenomenological analysis approach.

**Results:**

Data analysis resulted in the identification of three main topics: *fuzziness*, *domain specificity* and *dialogical dynamics* of reflection in professional practice. Reflection was conceptualized as a fuzzy process of contemplation and action, leading to change and hopefully improvement of personal performance and health care in general. Physicians’ experiences with reflection were different for the patient domain and the team domain. Whereas experiences in the patient domain were recalled first and discussed in relatively clear terms, those in the team domain came second and were discussed in more ambiguous terms. In order to achieve improvement in daily practice, honest and open dialogues were perceived as necessary. These dialogues were regarded as the result of an interplay between an internal and an external dialogue. The internal dialogue required sensitivity and courage of the individual; the external dialogue required psychological safety and encouragement of the environment. Within the team domain however, handling the external dialogue effectively was not self-evident, underlining the importance of psychological safety.

**Conclusions:**

This study draws attention to the interdependence between the individual and the collective contributions to reflective activity in professional practice. Apart from its importance to physicians’ individual medical performance, reflective activity is also important to the functioning of a team of physicians. To allow reflection to rise from an individual activity to a team activity, it is necessary to invest in a safe environment in which people are encouraged to think, act, and be engaged.

## Background

In the last decades, the dynamics within health care have changed: nowadays, physicians are confronted with knowledgeable patients, ever larger teams and increasing medical knowledge. As a result, the desired attributes of a ‘good doctor’ have evolved significantly, bringing concomitant change in the goals and expectations of the medical education field. For most of the twentieth century, the predominant focus of medical education across the professional continuum was the acquisition of and reflection upon medical knowledge and procedural skills. Professional practice today, however, requires skills that extend beyond mere medical knowledge, such as interpersonal communication, team building, management and leadership skills. This shift in attention toward generic skills has fueled the movement of competency-based medical education (CBME), in which all aforementioned skills have been clustered and translated into competencies [1–3]. It is widely acknowledged that an important mechanism for learning and improving this broad range of competencies and thus for becoming and staying a ‘good doctor’ is ‘reflection’ [4–7].

Since reflection is recognized as a crucial component of lifelong learning, there has been thoroughly invested in its theoretical understanding [8–10]. Reflection is often described as a cyclic process of analysis, asking questions and paraphrasing professional experiences or, as Finlay ([11], p. 1) states: “the point is to recapture practice experiences and mull them over critically in order to gain new understandings and improve practice accordingly”. In the context of CBME and lifelong learning, the focus of reflection is on developing the self by expanding one’s own knowledge base and interpretive capacity, and broadening one’s understanding of certain issues. This inward-looking and individual focus is thought to contribute to self-insight and self-improvement through analysis and understanding of specific learning experiences that teach knowledge, skills and attitudes [12–18]. Across the continuum of medical education and medical practice, reflection is documented in portfolios, checklists and protocols; also group reflection activities and one-on-one interaction between teachers/mentors and students/physicians are used to stimulate and assess reflection [19–22].

However, the way in which reflection has been taken up in medical education is problematic, as has been addressed in recent years, for instance by Ng et al. [12]. They call for caution with regard to reductionist approaches that break down reflection and its complexity into discrete components of activity, or steps of a process. In this way, reflection is approached in an overtly prescriptive way, i.e. reflection is conceptualized ‘as it should look like’, thereby establishing the link between using reflection as a tool to develop oneself and using it to assess individuals. Several authors, among whom Sumsion [23] and Rolfe [24], argue that reflection has been turned into a ‘tick box exercise’; the very essence of reflection may be compromised when it is enforced in an overly prescriptive manner, or when it is subjected to evaluation and reductive approaches.

Despite the situation as outlined above, within CBME literature, reflective capacity is discussed as an attribute of a competent professional and therefore physicians are expected to reflect on their performance. Since most continuous professional development (CPD) programs and quality assurance policies now include procedures or regulations to show some evidence of reflective practice, there is a growing need to develop new ways of thinking about reflection that recognize the complexities and relational qualities of today’s professional practice, without reducing reflection to a ‘product’ [25–27]. In order to inform reflective activities for practicing physicians, it is necessary to openly explore how physicians themselves describe their relation to reflection. In this study, we therefore addressed the following two interrelated research questions: (I) How do practicing physicians conceptualize reflection and (II) How do they experience reflective activity in their daily practice?

## Methods

### Study design

Being interested in understanding the phenomenon of reflection by physicians’ lived experiences, we chose an interpretative phenomenological research approach (IPA) [28, 29]. The IPA approach allowed us to investigate individual experiences and accounts of physicians while constructing an overall impression of their experiences with reflection in daily practice [30, 31]. We drew from the epistemological stance that people are self-interpretative beings, therefore knowledge is subjective and there is no ultimate truth [32]. According to this approach, it is only through a process of interpretation that meanings can be understood. It explicitly acknowledges and accepts the importance of the researchers’ interpretation and embraces the assumption that pre-understanding and co-creation by the researcher and the participants are what makes interpretations meaningful [29, 32]. Since we asked physicians to reflect on their reflections, the research itself is in fact a reflective activity.

Because the IPA approach is one of active engagement in which researchers bring their own backgrounds to the analytical process, practicing reflexivity is critical. In this reflexive spirit, we provide the following contextual information: the lead author (EB) has a background in art history and clinical neuropsychology; her collaborators have significant experience in studying medical education, but their own disciplinary backgrounds include teacher education (CV), medicine and philosophy (AS), biomedical science and competency-based education (SH) and healthcare policy and management (KL). Since these backgrounds might influence the approach to the subject, all researchers have been involved in the process of analysis.

### Setting and participants

We conducted this study in the Netherlands, where physicians are either employed by the hospital or organized in independent entrepreneur partnerships. Along the data collection, we invited hospital-based physicians from various specialties and (non-)academic institutions to participate. They were purposively sampled to provide a heterogeneous participant group in terms of medical specialty, age, and gender. Initially, we informed participants by email and telephone that the purpose of the interviews was to explore reflection. Subsequently, we invited them to consider participation. Upon acceptance of the invitation, we requested an individual consent. In total, 19 invitations were sent and all were received positively. However, six invitees were not able to take part because of agenda related reasons.

### Interviews and data collection

We held one pilot interview and 13 individual interviews. Interviewees were 8 men and 5 women from different age categories, representing 11 different specialties at 8 (academic and non-academic) hospitals (Table 1). In the interviews we focused on in-depth exploration, allowing the participants to talk freely without interference from others. An open ended interview guide was constructed based on our research questions. The interviewer (EB) imposed direction as little as possible, enabling participants to tell their own reflection story. She gave no specific introduction on reflection and the interview started with a so-called association question. Participants were invited to write down on post-its impressions, ideas and words coming to mind when thinking of ‘reflection’. Drawing on these connotations, we covered more specific items like feelings, thoughts and reactions, as well as concrete examples (‘Can you describe an actual situation in which you reflected? What was the start? How did the process look like? Where did it lead to?’) To close the interview, we invited participants to formulate a metaphor or transcending image of reflection. We conducted a pilot interview with the purpose of gathering feedback on the content of the initial interview guide as well as the interview technique. The first author (EB) conducted all interviews between March 2016 and November 2016. Interviews lasted approximately one hour and were conducted in the privacy of the physician’s office, whereas confidentiality was assured at the start of the interview. All interviews were audio-recorded and transcribed verbatim by a professional transcriptionist.

**Table 1:** Participants: gender, age, specialty and setting

Participant #	Gender and age	Specialty	Setting
1	Male in his 50s	Gynecology and obstetrics	Academic
2	Female in her 40s	Pediatrics	Academic
3	Female in her 60s	Internal medicine	Academic
4	Male in his 30s	Psychiatry	Non-academic
5	Male in his 40s	Trauma surgery	Academic
6	Female in her 40s	Neurology	Academic
7	Male in his 30s	ENT surgery	Academic
8	Male in his 50s	Clinical pathology	Non-academic
9	Female in her 40s	Radiology	Non-academic
10	Female in her 40s	Anesthesiology	Non-academic
11	Male in his 60s	Gastro-enterology	Academic
12	Male in his 40s	Surgical oncology	Non-academic
13	Male in his 50s	Neurology	Non-academic

### Data analysis

To deduce meaning from the data, the first author engaged in mind mapping as means to reflect on the participants’ reflections on reflection. Mind mapping involves creating visual diagrams with thought bubbles for brainstorming themes and drawing connections between them in a visual and non-linear way [33, 34]. Following each interview, a mind map was created in order to begin to track preliminary emerging themes, using the post-its as a starting point. First, the transcript was read to obtain a holistic view of the data as a whole and a naïve understanding was formulated. Second, the first author looked for ‘parts’ in the ‘whole’ by identifying passages, key words and phrases that elicited understanding about what the participant was saying about the concept and experience of reflection in daily practice. Third, these parts were categorized and - together with the author’s memos - converted into a mind map. Through constant comparative analysis, individual mind maps were accommodated in overarching mind maps. Saturation was reached at the point where interview 12 and 13 did not yield any new themes or input for recurring themes.

In order to establish credibility in the interpretation of data, mind maps were discussed within the research team and the emerging themes were compared to the original post-its and transcripts. More specifically, this process was shaped as follows: (i) initial understanding of the pilot interview was discussed with KL by re-listening interview fragments; (ii) initial understanding of interviews 1–2 was discussed with KL and SH by re-reading the transcripts; (iii) individual mind maps of interview 3–5 as well as an overarching mind map were discussed within the whole research team (CV, AS, SH, KL); (iv) individual mind maps of interview 6–9 as well as an overarching mind map was discussed with CV by re-reading transcripts; (v) an overarching mind map of interview 1–13 was discussed within the whole research team; a final comprehensive understanding was formulated by the first author in consultation with the research team.

## Results

### Overall results

Data analysis resulted in the identification of three main topics related to our two research questions: the *fuzziness*, *domain specificity* and *dialogical dynamics* of reflection in daily practice. In short, during the interviews reflection was conceptualized as an ongoing movement between contemplation and action orientation, leading to change and hopefully improvement of personal performance and health care in general. Physicians’ experiences with reflection were different for the patient domain and the team domain. Whereas reflection experiences in the patient domain were recalled first and discussed in relatively clear terms, reflection experiences in the team domain came second and were discussed in more ambiguous terms. In order to achieve improvement in daily practice, honest and open dialogues were perceived as necessary. These dialogues were regarded as the result of an interplay between an ‘internal dialogue’ or private conversation with oneself, and an ‘external dialogue’ or the act of sharing experiences with others. The internal dialogue required sensitivity and courage of the individual; the external dialogue required psychological safety and encouragement of the environment. We will now discuss these themes in detail below.

### Reflection as a fuzzy process

‘Reflection’ elicited process-related verbs, such as ‘becoming aware’, ‘holding still’, ‘looking back’, ‘observing and analyzing’ and ‘gaining insight’. Newly acquired insights were meant to ‘determine direction’ and led to outcomes such as ‘balanced conduct’, ‘quality improvement in own performance and health care in general’ and ‘ongoing learning’. Although the concept elicited these clear process-related verbs, participants also pointed to the fuzziness of the concept, stating that it was something ‘fuzzy’, ‘foggy’ and ‘difficult to grasp’, as Interviewee 3 observed:

*‘I think most people do understand what is meant by it, but that this is a concept that encompasses a lot of things.’*

Participants referred to a dialectical movement between contemplation and action or action orientation within the reflection process, as Interviewee 4 stated:

*‘If reflection does not lead to action or action-orientation, then it is like there is insight without movement. Then reflection stays in the atmosphere of associations, but it has to clench together and become clear, so that you can apply it to something. Because when something is cloudy, it disturbs and can deter. Reflection is like a process of rising above the fog: you seek clarity to get a view of the right direction and when you see the path ahead of you, you also want to go that direction, because it suits you.’*

### Domain specificity of reflection

Participants described a broad array of practice situations that triggered reflection. Reflection experiences that came to mind first were related to the patient domain; reflection experiences that seemed to be surrounded by more ‘fog’ were mostly related to the team domain and were reported in a later instance.

#### Patient domain

Overall, reflective activities that were mentioned first were closely tied to the medical domain, such as clinical reasoning, adaptive expertise and patient communication. Participants seemed to have little difficulty in finding the right words to describe how these reflection processes evolved. We interpreted this as a sign that participants felt most comfortable with reflection related to medical issues on the patient level. Reflection in these situations was associated with a state of concentration, as a means to assist the participant to pinpoint what was meaningful and of fundamental importance. Reflection was described as ‘slowing down’, ‘being on your guard’, ‘taking responsibility’ and ‘being open to other perspectives and possibilities yet standing your ground’. Reflection required effort, as other habits of mind might be more rewarding, such as speed or no-nonsense. All participants reported that the above mentioned elements lead them to ask themselves questions, such as ‘Did I miss things?’, ‘Did I close off too soon?’ or ‘How can I best ‘tune in’ on this patient and establish or restore a sense of connection?’ In the case of such questions, it was obvious to ask a colleague for help or discuss these issues during consultations with colleagues. As Interviewee 1 explained:

*‘In a consultation with peers, there is of course partial misunderstanding of each other's point of view, or people are not entirely well informed. It is exactly in that process where we analyze the whole case and sort out what happened exactly. Ultimately we reconstruct the story and then we can make a decision. Constructing this story is also very important in the sense that others can learn from it, because it will come to mind when they will come across a similar situation.’*

#### Team domain

Reflective activities that were only mentioned later on in the interviews were tied to communication issues with colleagues, team work and team dynamics, as participants started to reflect on aspects of reflection that are probably harder to describe. This cluster of situations did not only contain ad hoc situations that called for direct response, but also situations that seemed to develop gradually over time, such as an unequal division in tasks, responsibilities and participation within the team. With regard to these types of situations, participants seemed to experience more difficulty or ‘fog’ in describing how they navigated these situations and how reflection processes evolved. As Interviewee 4 explained:

*‘In your role toward the patient there are not that many barriers, you just do your work, it has to do with your medical competence. But there is also cooperation and communication with colleagues, those things. Some colleagues are very diplomatic in their communication, others are very direct. Well, it forces me to think about my own style, and in which situation I use which style.’*

The proportion of ‘fog’ in these reflection processes was tied to the degree of clarity about possible outcomes and the dependency on others. Interviewee 2 described her encounter with her new group of colleagues: ‘In new situations as these you just need to test the waters and see how the wind blows. You ask yourself, where do I fit in and what will be my role?’ In describing a similar situation, Interviewee 9 looked at it from a team perspective:

*‘When you enter a new team, you need to deal with this whole interaction, this group process. Who makes and who breaks the policy? Is there policy anyway? Where do we stand and where are we going? Have we thought about it at all?’*

This interviewee further widened her team perspective by relating her teamwork to the bigger picture of the organization:

*‘As a physician, you are a little cog in a very large whole and this can lead to a kind of indifference. I mean, you would like to change something but then you notice that the whole organization is inert and slow like syrup. Then you run the risk of developing this ‘every man for himself’ attitude and you just focus on your own little piece.’*

### Dialogical dynamics

From the participants’ reflection stories, reflection’s alternating movement between contemplation and action orientation seemed to unfold itself in an interplay between an internal dialogue (or private conversation with oneself) and an external dialogue (or actual conversation with others). These dialogues were triggered by situations in daily practice in both the patient domain as well as the team domain.

#### Internal dialogue

All participants noticed that a certain ‘emotional sensitivity’, ‘observational ability’ and ‘courage to have an honest look at oneself’ was conditional to start any internal dialogue. Interviewee 3 described this internal dialogue in the context of a patient contact:

*‘When I get irritated by a patient, and that just happens sometimes, I feel it inside. It happens quite often when someone shows claiming behavior. That is the moment that I think ‘hey, I need to pay attention now’. And yes, in that moment you should actually be able to take a step back and not give in to your feeling of irritation, although that may be your natural reaction. I know the moments in which I need to be extra alert and I also know what I can do to turn the tide.’*

The same interviewee also mentioned the importance of ‘taking a critical look at your own share’ in the case of a complication or medical error:

*‘You can say, ‘well, it was the other person, it has nothing to do with me, I don’t feel addressed or affected, I don’t feel responsible..’ But you can also think, what was my role in this whole story, that is what I mean with ‘taking a critical look at something’. Reflection just requires courage, because you also have to face your own imperfection and stupidity.’*

Interviewee 13 also mentioned that reflection was observing a situation, taking a step back and asking a question, instead of reacting based on your first impression. Ideally, the internal dialogue took place prior to the external dialogue:

*‘I think that over time, I try to understand things more by asking questions, instead of giving my opinion straight away.* [Interviewer: How did you learn that?] *Yes… through observations and by seeing how things happen, how people do things in conversations. My response also depends a lot on the moment and on the person: who is this person, do I know him or her better? Ideally you have some sort of repertoire at your disposal, like, you approach one colleague in this way and the other in that way..* [Interviewer: so before you start a conversation you have a conversation with yourself?] *Well, ideally yes, unless I am so angry that I cannot control myself, then I cannot find enough space for this conversation with myself.’*

#### External dialogue

Participants described that when a situation related to the patient domain or the team domain was discussed collectively, an external dialogue was initiated. In the case of a (dissatisfying) patient contact or lacking medical knowledge, participants frequently stated that they could easily call for help from a colleague. In the case of a complication or medical error, external dialogue was considered to be of vital importance for patient care, as Interviewee 12 mentioned:

*‘Talking to others about this is a possibility to unwind. Because you talk to each other about it, you can sort out how to prevent it from happening next time. And it creates this possibility form other members to say ‘you know, actually I experience the same thing’. So then you can see how you can improve practice as a group.’*

Other interviewees not only pointed to the importance of the external dialogue for direct patient care, but also for the team performance in the long run. As Interviewee 9 explained:

*‘If we as a group continue like this, we will get into trouble, eventually. Not so much in the sense that we don’t do well as individual medical specialists, but more in the sense that working together is challenging. Because you will get frustrations, because it seems like, well, over time you develop some sort of unequal division in tasks, responsibilities and participation and in discussion people keep aloof. And in our group there are quite some differences in characters and we just have to make it work.’*

Several participants mentioned the challenge of team performance and described how team issues could get out of hand, for example in the case of an approaching merger. Interviewee 8 described how team discussions got stuck because of a conflict of interest and he described the experiences with an external counselor:

*‘Then this external person does the reflection in your place, so to say. He is a kind of father figure and he can simply guide the group in this whole reflection process. He spells out the consequences and what you can or cannot expect from each other. And what choices there are and also the limitations of these choices, so that you get a clear picture again.’*

Interviewee 4 described his experiences with an approaching merger and mentioned that he individually looked for sparring partners outside his team:

*‘Sometimes I meet with peers outside my team to discuss the uncertainties about the merger. Like, how do I position myself? Am I too careful? Then you get tips, advice, feedback from colleagues who work elsewhere. So that is very good, yes. And because you do not work with these people directly, it is certainly safer to speak your mind.’*

#### Interplay of internal and external dialogue

Participants described an interplay between an internal and an external dialogue in daily practice. In the patient domain, discussing patient cases with others was recognized as a way of reflecting collectively; the tipping point between the internal and external dialogue was relatively straightforward and the process of collective reflection was clearly framed. In the team domain, however, the tipping point between the internal and external dialogue was less straightforward and the process of collective reflection was less clear because of ‘fuzzy’ team dynamics, issues related to the division of tasks and responsibilities, the sorting out of a shared group vision and an overall (diffuse) sense of psychological safety. The importance of this sense of security was reported by all participants. From the participants’ stories it became clear that the experience of psychological safety was a condition for the internal dialogue to be translated into an external dialogue. As Interviewee 5 explained:

*'In my department, we take a critical stance toward each other and sometimes my colleagues ask me about how I handled things in a surgical procedure. I know that people can just wonder and be curious, but where I work, it is like, you have done something and colleagues take this as an opportunity to rise above you, to display their knowledge to residents and nurses at the expense of you, that is just something I feel.'*

The importance of psychological safety also came to the fore in the case of a medical error, a situation that started in the patient domain and underwent a follow-up in the team domain. Although some participants noted that team discussions on these matters were meant as a possibility to ‘unwind’, not everyone felt safe enough to even report an error, because of the possibility of ‘becoming known as that person’. As Interviewee 8 mentioned:

*‘You just know that your own reaction to a medical error partly depends on how the other person reacts, to whom you must report. If you have some negative experiences with this, you are less inclined to report an error, while at the same time it is crucial for a good hospital setting.’*

From participants’ stories, the need for commitment and reciprocity arose. As Interviewee 3 talked about the teamwork with her residents, she characterized her attitude toward building trust, working together and supervising them as follows:

*‘I tell them in advance about me being a bit neurotic.. in the sense that I find it difficult to sort of let them go.. I tell them that if they feel that I am looking over their shoulder too much that they should blow the whistle. And I always ask honest feedback from them. By doing so, I hope that they will feel that as a supervisor, I am not so much standing above them, but next to them. Working together simply cannot be a one-way street.’*

## Discussion

In this study it was shown that physicians conceptualized reflection as a fuzzy process in which elements of contemplation and action alternated. Participants described this process as an interplay between an internal dialogue and an external dialogue. The sequence in which reflection experiences in daily practice were mentioned provided information about a layering in the experience of reflective activity. Participants first referred to reflection experiences in the patient domain (the context of physician-patient contacts and medical performance), which they discussed in relatively straightforward terms. In the sequence of the interview, participants also referred to reflection experiences in the team domain (the context of working together with other professionals), which they discussed in more ambiguous terms. From this finding we concluded that reflection experiences in the patient domain were more readily available than reflection experiences in the team domain. In the following section, we will elaborate on (i) the conceptualization of reflection as a two-fold activity on both the individual level as well as the collective level; (ii) the importance of reflection in the team domain to enable team development and foster improvement in the patient domain and (iii) the need to create psychological safety in the team domain as a condition for effective reflection.

Our findings indicate that physicians do not view reflection solely as a clear or straightforward attribute of the competent individual professional. When openly exploring, physicians conceptualize reflection as a hard to frame ‘fuzzy process’ that takes place both in the individual atmosphere – by means of an internal dialogue – as well as in the collective atmosphere – by means of an external dialogue. This view on reflection is somewhat contradictory to the view on reflection as a solidified individual competency, as expressed in the CBME literature [3–5, 12, 19, 25]. In CBME literature, reflective capacity is portrayed as an individual possession, an ability to engage in reflective activity which can be acquired. This contrast between reflection viewed as an individual attribute and reflection viewed as something that commutes between the individual and his/her social environment resonates with the work of Lingard about individualist and collectivist discourses of medical competence. As described by Lingard, the individualist discourse views competence as a construct which individuals acquire and possess, which is context-free and which represents a state to be achieved. In the collectivist discourse, competence evolves from participation in authentic situations, is distributed across networks of persons and manifests itself in interconnected behaviors [35, 36]. If we consider the findings of the current study in the light of these individualist and collectivist discourses, then the notion of reflection requires expansion from ‘self-reflection’ to ‘collective reflection’. This collective dimension of reflection has been described by several authors in communication and learning sciences, emphasizing the role of sharing experiences for the purpose of both individual learning as well as collective learning [37–39]. As Boud [40] states, “reflection in collective settings cannot be an individual act if it is to influence work that takes place with others.” Reflection on one’s individual process or the team process can be facilitated either in a group of physicians or in a one-on-one interaction between a physician and peer or a facilitator. By explicating preliminary individual understandings and sharing individual experiences with others, a shared understanding about work practices and work-related experience is established and collective learning can emerge. Outcomes of this process of sharing and joint reflection, either during a one-on-one or a group session, can prompt both individual learning as well as collective learning.

Our analysis rendered two layers of reflection experiences, first related to the patient domain and second related to the team domain. Although distinguishable from each other, these two layers were closely connected to each other. For example, the situation of an approaching merger, which could cause disturbance within the team, could eventually impact the way care was organized and provided for in the patient domain. Collective reflection in the team domain could be a powerful method to ensure that team members learn what is necessary to remain motivated individually and as a team, to solve work-related problems collectively, to adequately discuss tensions among team members and to make group decisions that can count on sufficient support from all members. In communication and learning sciences this is defined as collective learning: the joint creation of collective rules, procedures, norms and values that determine the behavior of the group of team members [41]. Collective learning also implies that members regularly take time to reflect together and, if necessary, adjust the working method based on experiences and new insights. This ongoing process of reflecting together on common concerns means that the collaboration within the team is constantly improving, so that the team can maintain a high level of functioning when circumstances change [41, 42]. This calls for a broadening of the reflective focus of team members by concentrating on both their individual and collective part in team work. Since team work will always be a challenge, the question remains as to how collective reflection can be handled properly: by increasing openness, honesty and space for feedback. This brings us to our last point, the importance of psychological safety.

From the findings it became clear that reflecting together and sharing experiences with others is only possible if there is a certain degree of psychological safety. Only when team members feel safe, they will be willing to take interpersonal risks, be vulnerable and give feedback to colleagues without being afraid that this might have negative consequences for the relationship with team members [43]. When a psychologically safe environment is lacking, team members would prefer to remain silent, especially when it concerns learning from mistakes (as is the case with medical errors) [44, 45]. In addition, a sense of joint responsibility and commitment and the extent to which someone identifies with the team is important [43]. Our findings suggest that reflecting together with team members on team issues is not self-evident; participants noticed that – in some matters – they preferred to speak with people outside their own ‘working bubble’. Participants also noticed that in team discussions colleagues might keep aloof and engage in abstract conversation as an alternative to action that meets the cultural norm. Possible consequences of this state of affairs may be mutual tension or development of an ego centric attitude, thereby possibly undermining performance in its broadest sense [43]. Creating a climate of psychological safety, therefore, is of central importance for fostering open dialogues, thereby fueling both individual and team learning and development.

### Limitations

As with any qualitative study, a conducive partnership between the researcher and participant is necessary for rendering a thorough insight into the topic of interest. For this purpose, not only the interviewer’s listening sensitivity is important, but also participants’ abilities to verbalize their experiences. Although all participants’ accounts of their conceptualization and experience of reflective activity in daily practice are unique, IPA methodology requires that the researcher looks for themes that are shared by all participants [30]. Once identified, the themes may shape the direction of the analysis, rendering IPA vulnerable to researcher bias. As we were mindful of this issue, we took steps to minimize such bias, primarily through ongoing discussions among the authors to verify our coding scheme, interpretations and conclusions. Apart from this methodological consideration, it should also be noted that all participants were hospital-based physicians working within the Dutch healthcare system. Therefore, it is not self-evident that the findings of the current study are transferable to physicians outside the Netherlands, or to physicians outside the hospital setting, such as general practitioners. Caution is also required when it comes to transferability of the findings to other groups of healthcare professionals, such as nurses.

### Implications for practicing reflection

In the wake of previous studies, this study suggests to explore reflection as a collective activity affecting the larger health profession team or organization, rather than approaching reflection as a solitary, purely introspective activity. In the context of continuous professional development (CPD) programs, quality assurance policies and re-registration procedures, dedicated time should be provided for physicians to reflect on both their individual process as well as on common concerns within the team. Written reflective activities can be used, but based on the results of the current study, a reflection format in which an important role is reserved for the dialogical aspects of reflection could be more fitted to practice. Reflection could be facilitated in meetings following the completion of a multisource feedback (MSF) round within the team [46]. These meetings could either be one-on-one meetings with a facilitator or peer or team meetings led by a facilitator. In view of the importance of psychological safety, the use of skilled facilitators would be desirable. The team discussion with peers may also contribute to a sense of solidarity and mutual respect, since previous research has shown that sharing personal reflections with peers could improve the quality of collegial relationships and heighten the chance of performance improvement [47].

### Recommendations for future research

Future research should continue to focus on reflection related to both the individual as well as the team functioning of physicians and the mutual influence of these two. As noted above, reflection on the physician’s individual professional development or the team process can be facilitated in both one-on-one meetings between a physician and a peer or facilitator, or in group meetings led by a facilitator. Further research into the impact and perceived usefulness of different strategies to facilitate reflection in these meetings is warranted. It is known from previous research that guided reflection with the help of a facilitator helps to achieve performance improvement. However, it is unclear as to whether or how the presence of colleagues in reflection meetings to debrief individual performance feedback impacts on the process or its outcomes. Furthermore, investments must be made to develop and validate tools that can be used to evaluate the collective performance of groups of physicians.

## Conclusions

The contribution of this reflection study is twofold: a majority of studies deals with the theoretical aspects of reflection and are prescriptive, conceptualizing what reflection should look like, whereas the current study starts with the practitioners themselves and maps how these practitioners describe their own relation to reflection. Secondly, it confirms some of the points that have been described in the reflection literature, for instance that reflection is an ambiguous concept which can have benefits even when it does not lead to improved practice. Physicians experienced reflection as an activity related to their medical performance, but also as an activity related to their performance within the team to which they belong. In realizing open dialogues with colleagues on the team level, psychological safety plays an important role. Both the individual physician as well as the team of physicians and other health professionals have a responsibility in the development of abilities such as listening, sensitivity and community spirit. We suggest that attention to these aspects will foster synergy between reflective physicians and will culminate in reflective teams.

## Abbreviations

CBME: Competency-Based Medical Education
CPD: Continuing Professional Development
MSF: multisource feedback

## References

[1] Institute of Medicine (2003). Health professions education: a bridge to education.

[2] Frenk J, Chen L, Bhutta ZA, Cohen J, Crisp N, Evans T, Fineber H, Garcia P, Ke Y, Kelley P, Kistnasamy B, Meleis A, Naylor D, Pablos-Mendez A, Reddy S, Scrimshaw S, Sepulveda J, Serwadda D, Zurayk H (2010). Health professionals for a new century: transforming education to strengthen health systems in an interdependent world. Lancet.

[3] Carraccio CL, Englander R (2013). From Flexner to competencies: reflections on a decade and the journey ahead. Acad Med.

[4] Sandars J (2009). The use of reflection in medical education: AMEE guide no. 44. Med Teach.

[5] Mann K, Gordon J, MacLeod A (2009). Reflection and reflective practice in health professions education: a systematic review. Adv Health Sci Educ.

[6] Epstein RM (2008). Reflection, perception and the acquisition of wisdom. Med Educ.

[7] Mamede S, Schmidt HG (2004). The structure of reflective practice in medicine. Med Educ.

[8] Kinsella EA (2010). Professional knowledge and the epistemology of reflective practice. Nurs Philos.

[9] Nguyen QD, Fernandez N, Karsenti T, Charlin B (2014). What is reflection? A conceptual analysis of major definitions and a proposal of a five-component model. Med Educ.

[10] Clarà M (2015). What is reflection? Looking for clarity in an ambiguous notion. J Teach Educ.

[11] Finlay L. Reflecting on reflective practice. PBPL paper 52. 2008. (http://www.open.ac.uk/opencetl/resources/pbpl-resources/finlay-l-2008-reflecting-reflective-practice-pbpl-paper-52). Accessed 14 Dec 2017.

[12] Ng SL, Kinsella EA, Friesen F, Hodges B (2015). Reclaiming a theoretical orientation to reflection in medical education research: a critical narrative review. Med Educ.

[13] Carr SE, Johnson PH (2013). Does self-reflection and insight correlate with academic performance in medical students?. BMC Med Educ.

[14] Stephens MB, Reamy BV, Anderson D, Olsen C, Hemmer PA, Durning SJ, Auster S (2012). Writing, self-reflection, and medical school performance: the human context of health care. Mil Med.

[15] Ballon BC, Skinner W (2008). “Attitude is a little thing that makes a big difference”: reflection techniques for addiction psychiatry training. Acad Psychiatry.

[16] Das Gupta S, Charon R (2004). Personal illness narratives: using reflective writing to teach empathy. Acad Med.

[17] Stark P, Roberts C, Newble D, Bax N (2006). Discovering professionalism through guided reflection. Med Teach.

[18] Nevalainen MK, Mantyranta T, Pitkala KH (2010). Facing uncertainty as a medical student—a qualitative study of their reflective learning diaries and writings on specific themes during the first clinical year. Patient Educ Couns.

[19] Hall P, Byszewski A, Sutherland S, Stodel EJ (2012). Developing a sustainable electronic portfolio (ePortfolio) program that fosters reflective practice and incorporates CanMEDS competencies into the undergraduate medical curriculum. Acad Med.

[20] Whitehead C, Selleger V, Kreeke J, Hodges B (2014). The ‘missing person’ in roles-based competency models: a historical, cross-national, contrastive case study. Med Educ.

[21] Paul R, Beamish AJ, Suter VE, Ruprai CK, Al-Muzaffar L, Jiraporncharoen W (2013). Assessing reflective practice. Educ Prim Care.

[22] Wald HS, Borkan JM, Taylor JS, Anthony D, Reis SP (2012). Fostering and evaluating reflective capacity in medical education: developing the REFLECT rubric for assessing reflective writing. Acad Med.

[23] Sumsion J, Fleet A (1996). Reflection: can we assess it? Should we assess it?. Assess Eval High Educ.

[24] Rolfe G (2014). Rethinking reflective education: what would Dewey have done?. Nurse Educ Today.

[25] Batalden P, Leach D, Swing S, Dreyfus H, Dreyfus S (2002). General competencies and accreditation in graduate medical education. Health Aff.

[26] Holmboe ES, Foster TC, Ogrinc G (2016). Co-creating quality in health care through learning and dissemination. J Contin Educ Health..

[27] Kotzee B (2012). Private practice: exploring the missing social dimension in ‘reflective practice’. Stud Cont Educ.

[28] King N, Carroll C, Newton P, Dornan T (2002). “You can’t cure it so you have to endure it”: the experience of adaptation to diabetic renal disease. Qual Health Res.

[29] Finlay L, Ballinger C (2006). Qualitative research for allied health professionals: challenging choices.

[30] Starks H, Brown TS (2007). Choose your method: a comparison of phenomenology, discourse analysis, and grounded theory. Qual Health Res.

[31] Bunniss S, Kelly DR (2010). Research paradigms in medical education research. Med Educ.

[32] Wojnar DM, Swanson KM (2007). Phenomenology: an exploration. J Holist Nurs.

[33] Buzan T, Buzan B (2003). The mind map book: radiant thinking – major evolution in human thought.

[34] Tattersall C, Powell J, Stroud J, Pringle J (2011). Mind mapping in qualitative research. Nurs Times.

[35] Lingard L, Lingard L, Hodges B (2012). Rethinking competence in the context of teamwork. The question of competence: reconsidering medical education in the twenty-first century.

[36] Lingard L (2016). Paradoxical truths and persistent myths: reframing the team competence conversation. J Contin Educ Health.

[37] Reynolds M, Vince R (2004). Organizing reflection.

[38] Senge P (2006). The fifth discipline: the art and practice of the learning organization.

[39] Van Woerkom M, Croon M (2008). Operationalising critically reflective work behavior. Pers Rev.

[40] Boud D, Bradbury H, Frost N, Kilminster S, Zukas M (2010). Relocating reflection in the context of practice. Beyond reflective practice. New approaches to professional lifelong learning.

[41] Edmonson A, Kramer R, Cook K (2004). Psychological safety, trust, and learning: a group-level lens. Trust and distrust in organizations: dilemmas and approaches.

[42] Gibb JR, Leland LP, Gibb JR, Benne KD (1964). Climate for trust formation. T-group theory and laboratory method.

[43] Edmonson A (1999). Psychological safety and learning behavior in work teams. Adm Sci Q.

[44] Carmeli A, Hoffer GJ (2009). High-quality relationships, psychological safety, and learning from failures in work organizations. J Organ Behav.

[45] Edmonson A. Learning from failure in health care: frequent opportunities, pervasive barriers. Qual Saf Health Care. 2004;13. Suppl 2;ii3-ii9. 10.1136/qshc.2003.009597 PMC1765808 15576689

[46] Sargeant J, Lockyer J, Mann K, Holmboe E, Silver I, Arson H, Driessen EW, MacLeod T, Yen W, Ross K, Power M (2015). Facilitated reflective performance feedback: developing an evidence- and theory-based model that builds relationship, explores reactions and content, and coaches for performance change (R2C2). Acad Med.

[47] Overeem K, Driessen EW, Arah OA, Lombarts MJMH, Wollersheim HC, Grol RPTM (2010). Peer mentoring in doctor performance assessment: strategies, obstacles and benefits. Med Educ.

